# Anti-tumour effects of antibodies targeting the extracellular cysteine-rich region of the receptor tyrosine kinase EphB4

**DOI:** 10.18632/oncotarget.3199

**Published:** 2015-03-25

**Authors:** Sally-Anne Stephenson, Evelyn L. Douglas, Inga Mertens-Walker, Jessica E. Lisle, Mohanan S.N. Maharaj, Adrian C. Herington

**Affiliations:** ^1^ Institute of Health and Biomedical Innovation, Queensland University of Technology, Translational Research Institute, Princess Alexandra Hospital, Queensland, Australia; ^2^ Australian Prostate Cancer Research Centre-Queensland, Queensland University of Technology, Translational Research Institute, Princess Alexandra Hospital, Queensland, Australia; ^3^ Faculty of Health, Queensland University of Technology, Brisbane, Queensland, Australia; ^4^ The Queen Elizabeth Hospital, University of Adelaide, Adelaide, South Australia, Australia

**Keywords:** EphB4, receptor tyrosine kinase, monoclonal antibody therapy, anti-cancer

## Abstract

EphB4 is a membrane-bound receptor tyrosine kinase (RTK) commonly over-produced by many epithelial cancers but with low to no expression in most normal adult tissues. EphB4 over-production promotes ligand-independent signaling pathways that increase cancer cell viability and stimulate migration and invasion. Several studies have shown that normal ligand-dependent signaling is tumour suppressive and therefore novel therapeutics which block the tumour promoting ligand-independent signaling and/or stimulate tumour suppressive ligand-dependent signaling will find application in the treatment of cancer. An EphB4-specific polyclonal antibody, targeting a region of 200 amino acids in the extracellular portion of EphB4, showed potent *in vitro* anti-cancer effects measured by an increase in apoptosis and a decrease in anchorage independent growth. Peptide exclusion was used to identify the epitope targeted by this antibody within the cysteine-rich region of the EphB4 protein, a sequence defined as a potential ligand interacting interface. Addition of antibody to cancer cells resulted in phosphorylation and subsequent degradation of the EphB4 protein, suggesting a mechanism that is ligand mimetic and tumour suppressive. A monoclonal antibody which specifically targets this identified extracellular epitope of EphB4 significantly reduced breast cancer xenograft growth *in vivo* confirming that EphB4 is a useful target for ligand-mimicking antibody-based anti-cancer therapies.

## INTRODUCTION

EphB4 is a member of the largest family of receptor tyrosine kinases and is an important regulator of fundamental physiological and pathophysiological processes such as tissue patterning during development, angiogenesis and tumour progression [1]. Despite significant promiscuity between other Eph family members and the ephrin ligands, the single physiologically-relevant ligand of the EphB4 receptor is ephrin-B2 [2]. Both the receptor and the ligand are membrane-bound but usually expressed on neighbouring cells. Forward signaling, through EphB4, and reverse signaling, through ephrin-B2, requires heterotetramerisation of two receptors on one cell with two ligands on a neighbouring cell [3]. This normal interaction between EphB4 and ephrin-B2 therefore requires direct cell-cell contact and induces forward signaling of the receptor that leads to tumour suppression and reverse signaling through the ligand that stabilizes cell-cell adhesion and can stimulate angiogenesis if the ephrin-B2 expressing cell is an endothelial cell [3].

Changes to the normal Eph-ephrin balance, for example to high EphB4 and low ephrin-B2, disrupts normal ligand-dependent signaling and promotes ligand-independent-mediated mechanisms that drive tumourigenesis [4–5]. Most commonly, the EphB4/ephrin-B2 balance in many cancer cells is disrupted by over-expression of the EphB4 receptor. EphB4 is reported as over-expressed in many epithelial cancers including, but not limited to, prostate (66% of cases studied) [6–7], colon (63–100%) [8–10], breast (58–94%) [11–15], ovarian (80–100%) [16–18], endometrial (100%) [19], pancreatic (100%) [20], lung (100%) [21] and cervical cancers (95%) [22–23]. Combining the data from these studies, currently to date, EphB4 protein levels have been tested in 1318 individual tumour samples and is increased above the level in normal matched tissues in 1046 (82%) of these. The mechanisms that drive EphB4 over-expression in cancer cells have not been determined although EphB4 itself was recently reported to regulate the estrogen receptor α and vimentin in breast cancer [24–25]. Amplification of the EphB4 gene has also been reported in clinical samples and cell lines from several cancer tissues including prostate, breast, bladder and head and neck [6, 11, 26–27].

The critical importance of EphB4 in tumour progression is demonstrated by studies using over-expression and knockdown strategies [6, 11, 28]. Forced over-expression of EphB4 in non-tumorigenic MCF10A breast cells and in 22Rv1 prostate cancer cells led to transformation of the MCF10A line and increased the metastatic phenotype of the 22Rv1 cells [28]. Addition of soluble clustered ligand abrogated these effects supporting a hypothesis that over-expression of EphB4 activates ligand-independent tumour promotion pathways and that ephrin-B2 ligand stimulation is tumour suppressive. Correspondingly, knockdown of EphB4 in several cancer cell lines consistently resulted in a 70–80% reduction in cancer cell viability, an 8–16 fold increase in apoptosis and up to an 80% reduction in cell migration and invasion [6, 11]. Furthermore, *in vivo* experiments targeting xenograft tumour cells expressing EphB4 using anti-sense oligonucleotides and monoclonal antibodies have demonstrated significant inhibition of tumour growth [6, 10, 11, 16].

EphB4/ephrinB2 bidirectional signaling has an established role in the formation of the vascular system, as evidenced by embryonic lethality in knockout mouse studies due to malformed vascular architecture [29–30] and functional experiments that show the critical requirement for bidirectional signaling for arteriovenous differentiation [31–32]. Given the importance of angiogenesis to tumour growth, several groups have explored the roles of EphB4 in this process, many using over-expression strategies to reconstruct or block either forward or reverse signaling with signaling defective mutants, soluble extracellular domain proteins, antibodies or small molecule inhibitors [33–38]. Tumour cells expressing dominant negative EphB4 incapable of forward signaling but able to stimulate ephrin-B2 reverse signaling, attracted endothelial cells, stimulating cell invasion, survival and proliferation and this correlated with tumours with larger blood vessels and a higher blood content [33]. Soluble monomeric EphB4 can block tumour angiogenesis and is being explored as anti-tumour therapeutics [34–35]. Similarly, antibodies that target ephrin-B2 and the extracellular fibronectin type III domains of EphB4 have been shown to modulate angiogenesis and inhibit tumour growth by mechanisms that are still unclear [36–37].

We used a peptide exclusion approach to identify an epitope within the extracellular cysteine-rich domain of EphB4 that is targeted by a commercially available polyclonal antibody with *in vitro* anti-cancer effects [39]. A panel of antibodies raised to a peptide including this epitope also show similar anti-cancer effects, including inhibition of the growth of established tumours and reduction of tumour mass. These studies have established EphB4 as a key target for the development of new anti-cancer therapies to which significant effort should be directed.

## RESULTS

### Validation of the H200 anti-EphB4 polyclonal antibody

**Figure 1:**
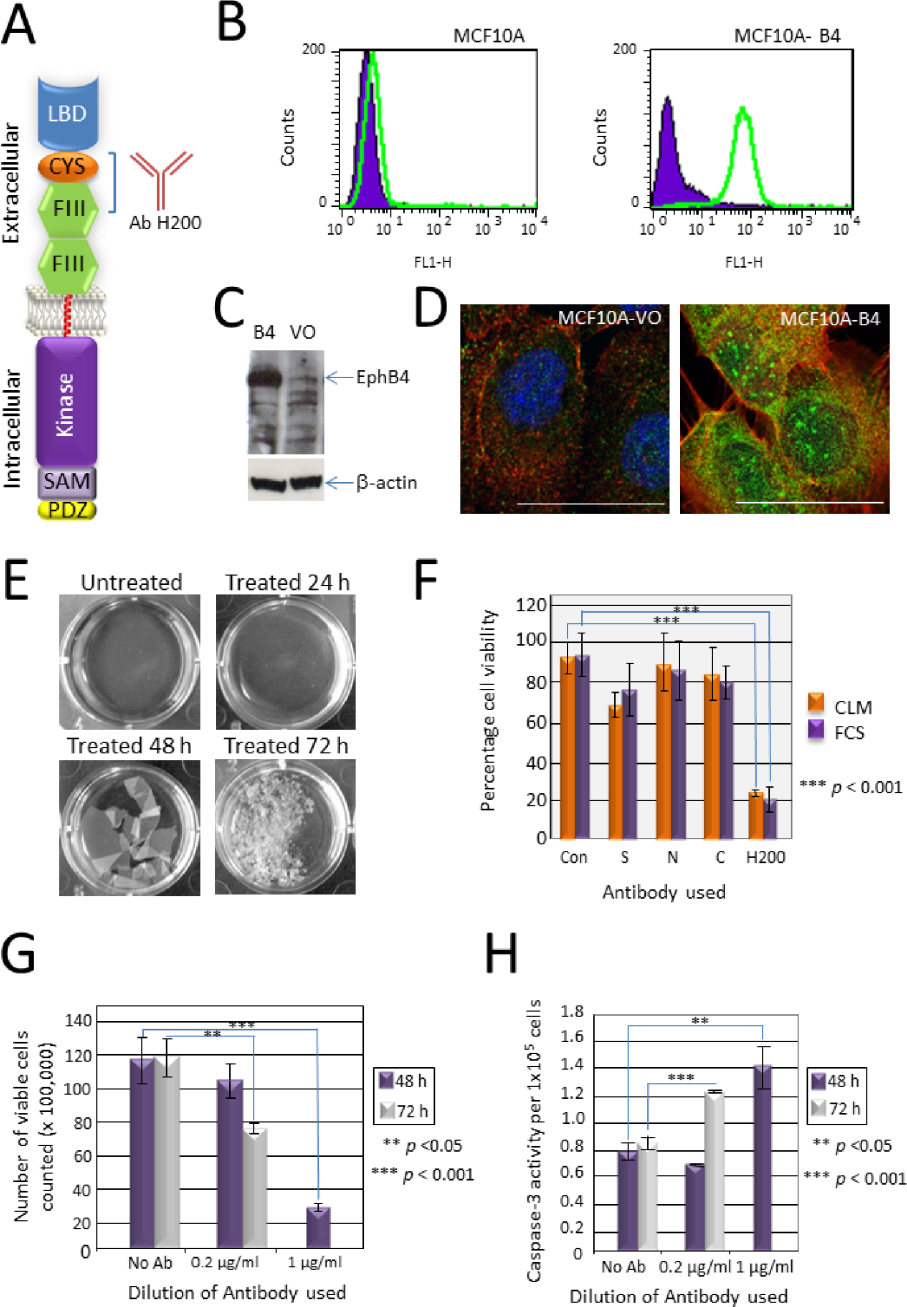
Validation of the EphB4-specific antibody H200 and its effect on cancer cell growth *in vitro* **(A)** The H200 antibody recognizes a 200 amino acid sequence in the extracellular domain of EphB4 spanning the cysteine rich domain (CYS) and the first fibronectin type III repeat (FIII). LBD—globular ligand binding domain, SAM—sterile alpha motif, PDZ—PDZ domain. **(B)** Flow cytometry comparing parental MCF10A cells with EphB4 over-expressing MCF10A-B4 cells using the H200 antibody showing an increase in fluorescence of the EphB4 over-expressing cells. **(C)** Western blot analysis with the H200 antibody identifying EphB4 over-expressed in the MCF10A-B4 cells (B4) compared with the parental empty vector only MCF10A-VO cells. β-actin was used to demonstrate equal loading. **(D)** Immunofluorescence using the H200 antibody to identify EphB4 (green) over-expressed in the MCF10A-B4 cells compared to the parental cells transfected with the empty vector (VO). Phalloidin-TRITC (red) stains F-actin, DAPI (blue) stains the nucleus. Co-localisation of EphB4 and F-actin (yellow). Bar = 10 μm. **(E)** Photomicrographs of cells treated with 0.4 μg/ml H200 antibody added to confluent monolayers of SW480 colon cancer cells for different lengths of time. **(F)** Trypan blue assay of SW480 cells treated with four different EphB4-specific antibodies for 48 h. Antibodies included the Ziemiecki lab rabbit polyclonal antibody (S), EphB4 (N-19) (N), EphB4 (H-200) (H) and EphB4 (C-16) (C) all from Santa Cruz Biotechnology. The H200 antibody showed a reduction in the number of viable cells compared to the control untreated (Con) and the other three antibodies irrespective of the presence (FCS) or absence (CLM) of complement. **(G)** Trypan blue assay of SW480 cells treated with either 0.2 μg/ml or 1 μg/ml H200 for 48 or 72 h. A significant reduction in the number of viable cells was seen at 72 h with the low concentration (*p* < 0.05) and at 48 h with the higher concentration (*p* < 0.001), with no viable cells remaining after 72 h treatment with 1 μg/ml H200. **(H)** Caspase-3 assay of SW480 cells treated with different concentrations of H200 (as per Figure 1G). The reduction in cell viability correlates with an increase in caspase-3 activity, statistically significant at 72 h with 0.2 μg/ml (*p* = 0.002) and at 48 h with 1 μg/ml (*p* = 0.003).

The commercial H200 polyclonal antibody (Santa Cruz) was raised to a 200 amino acid sequence in the extracellular domain of human EphB4 that spans the cysteine-rich region and the first fibronectin type III repeat (Figure 1A). To confirm that this antibody can identify EphB4 we compared the non-transformed breast cell line MCF10A which expresses a low endogenous level of EphB4 with MCF10A cells engineered to over-express full length EphB4 (MCF10A-B4) using three different techniques. Initially cell surface expression of EphB4 in both the parental and derivative was compared by flow cytometry. A clear increase in fluorescence of the MCF10A-B4 when compared with the MCF10A cells (green peak shifted to right) shows that the H200 polyclonal antibody (Ab) is binding to surface expressed EphB4 in the MCF10A–B4 cells (Figure 1B). The H200 pAb was then used in Western blot analysis using total protein isolates extracted from both cell lines. A strongly immunoreactive band at the predicted molecular weight of EphB4 (120 kDa) was detected with the H200 antibody in the sample from the MCF10A-B4 cells compared to low expressing control empty vector only MCF10A-VO cells (VO) (Figure 1C). Finally, EphB4 was detected in MCF10A-VO and MCF10A-B4 cells using immunofluorescence (Figure 1D). The increased green fluorescence in the MCF10A-B4 cells when compared with MCF10A-VO cells indicates that the H200 pAb recognizes over-expressed EphB4 protein. Together these results show that the H200 pAb recognizes EphB4.

### H200 pAb targets extracellular EphB4 and causes the death of tumour cells grown *in vitro*

H200 pAb was added to confluent monolayers of the EphB4 positive colon cancer cell line SW480. After 24 h, the edge of the confluent monolayer had separated from the bottom of the tissue culture well (Figure 1E). At 48 h the sheet of cells was completely lifted and gentle agitation caused the fragile sheet to begin to break apart. By 72 h cells were present either separately or in small clusters and > 80% stained with trypan blue indicating cell death. To determine if this was a specific response of the cells to the H200 antibody, three other antibodies targeting EphB4 were also tested. In this assay, treatment with H200 reduced cell viability to approximately 20% (*p* = 0.0006) whereas treatment with the Swiss pAb (S) reduced cell viability to approximately 70% (*p* = 0.006) (Figure 1F). No significant effect was seen after addition of the other Santa Cruz polyclonal antibody preparations that target different regions of the EphB4 protein (N-19 targets an N-terminal extracellular epitope and C-16 targets a C-terminal intracellular epitope) (Figure 1F). This suggests that this H200-induced decreased viability response is mediated through an epitope in the extracellular part of EphB4 recognised by antibodies in the H200 polyclonal antibody preparations. To test whether the H200 response was complement-mediated, heat-inactivated fetal calf serum (complement limited medium (CLM)) was compared with medium containing 10% complement-active normal fetal calf serum (FCS). The antibody effect was the same in both conditions showing that complement does not play a role in this response (Figure 1F).

### The H200-mediated tumour cell death response is dose-dependent and induces apoptosis

To determine the mode of cell death caused by the H200 antibody, confluent SW480 cells were incubated with two different dilutions of H200 antibody (final concentrations 1 μg/ml and 0.2 μg/ml) and viability was again assessed at two different time points (48 h and 72 h) using a trypan blue exclusion assay. Increasing concentrations of antibody resulted in more cells staining with trypan blue, suggestive of a dose-dependent antibody cell death response (Figure 1G). There were no viable cells after treatment with 1 μg/ml H200 for 72 h. To determine the general mechanism of cell death, we used both a caspase-3 activity assay as an indicator of apoptosis and an LDH activity assay as a measure of general cell toxicity. A concomitant and significant increase in caspase-3 activity, with no change in LDH activity suggests that the mechanism of cell death was through induction of apoptosis (Figure 1H and data not shown).

### EphB4-specific H200 broadly targets EphB4-positive tumour cells

**Figure 2:**
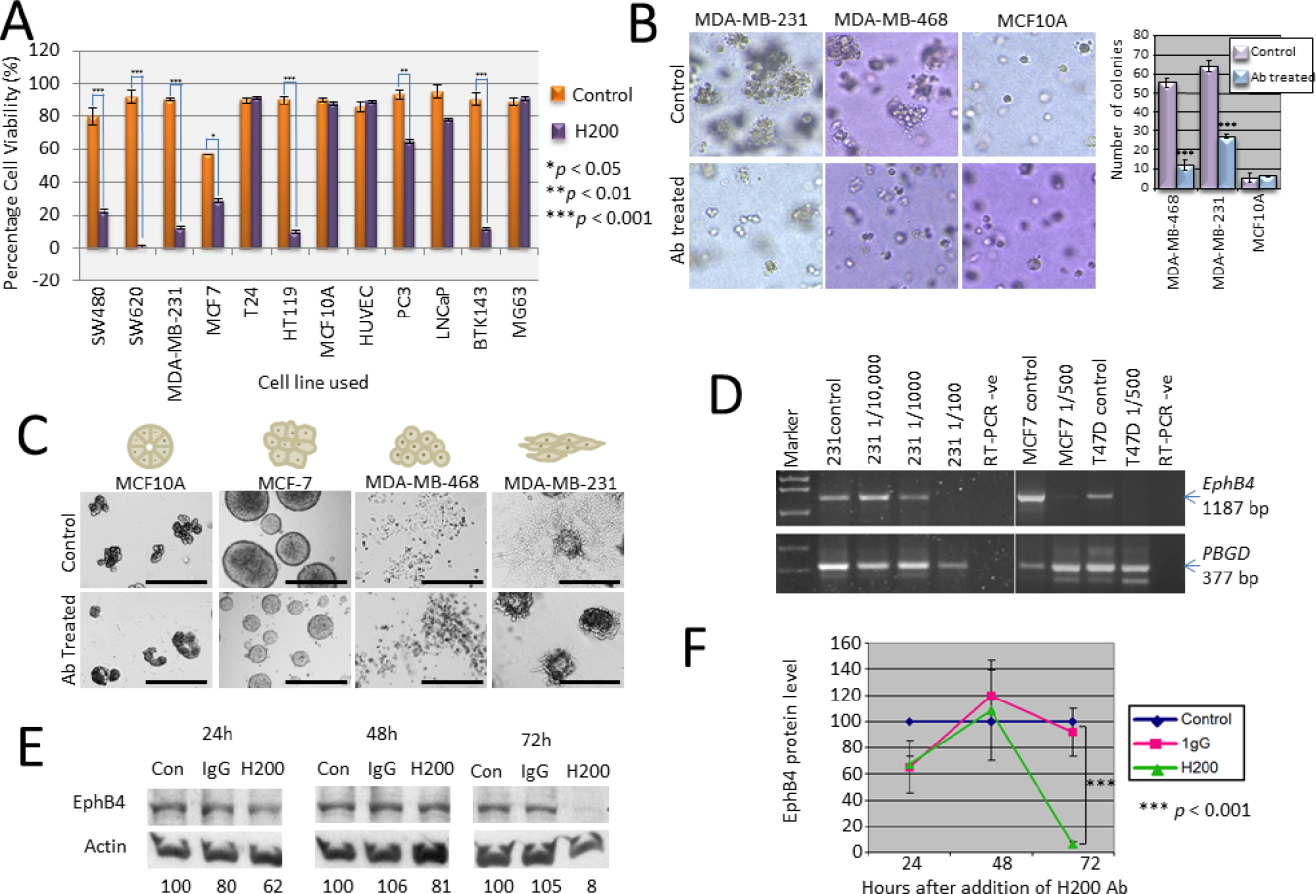
Effects of H200 pAb treatment on *in vitro* cell growth characteristics, gene expression and protein levels **(A)** The H200 pAb was tested using several different cell lines of colon (SW480, SW620), breast (MDA-MB-231, MCF-7, MCF10A), bladder (T24, HT119), endothelial (HUVEC), prostate (PC3, LNCaP) and osteosarcoma (BTK143, MG63) origins and after 48 h a significant reduction in the number of viable cells, determined using a trypan blue assay, when compared with untreated control cells was seen for all but T24, MCF10A, LNCaP, HUVEC and MG63. **(B)** Antibody targeting of EphB4 inhibits tumour cell colonisation of soft agar. MDA-MB-231, MDA-MB-468 and MCF10A cells were suspended in soft agar in the presence of EphB4 antibody (10 μg/ml) (Ab treated) or PBS vehicle control (Control) administered at the time of suspension. Colonies of more than 5 cells were counted from four different low power fields and the results are represented graphically. **(C)** Treatment with H200 prevented tubular nework formation on Matrigel. MDA-MB-468, MDA-MB-231, MCF-7 and MCF10A cells were plated on Matrigel in the presence of EphB4 antibody (10 μg/ml) (Ab treated) or PBS vehicle control (Control) and images representative of each assay were taken at 20× magnification after 4 days. **(D)** RT-PCR analysis of *EphB4* gene expression (PCR product = 1187 bp) in breast cancer cell lines treated with antibody or untreated (control). Expression of the *PBGD* housekeeping gene (377 bp) was used as a control for the integrity of the cDNA and relative comparison of *EphB4* expression levels. M1 = pUC19/*Hpa*II marker, M2 = Lambda/*Hind*III. **(E)** Western blot analysis showing that treatment with H200 causes loss of EphB4 protein at 72 h. β-actin was detected as a loading control and the total amount of EphB4 protein normalized to this using densitometry (values expressed as percentage of control). **(F)** Graph showing effect of H200 antibody on total EphB4 levels (relative to control) with a significant reduction seen at 72 h (*p* = 0.0001).

To confirm that the effect seen in SW480 cells after addition of the H200 antibody was not specific to this colon cancer cell line only, the experiments using H200 were repeated using confluent monolayers of various cancer cell lines including colon cancer cell line (SW620), breast cancer cell lines (MDA-MB-231, MCF-7), bladder cancer cell lines (T24, HT119), prostate cancer cell lines (PC3, LNCaP), the non-transformed breast cell line MCF10A, primary human umbilical vein endothelial cells (HUVEC) and osteosarcoma cell lines (BTK143, MG63). Significantly decreased viability was seen in most cancer cell lines after antibody treatment with the exception of T24, LNCaP and MG63 (Figure 2A). The non-transformed breast line MCF10A and normal HUVEC, both EphB4 positive [32, 40], were also unaffected (Figure 2A).

### The EphB4 antibody inhibits anchorage independent growth and tubular network formation of cancer cells

The ability to form colonies in soft agar (anchorage-independent) and tubular network formation on Matrigel are sensitive indicators of metastatic potential for cancer cell lines [41–42]. MCF10A breast epithelial cells and breast cancer cell lines MDA-MB-231 and MDA-MB-468 were grown suspended in soft agar. Addition of the Ab significantly (*p* < 0.001) inhibited the number of colonies formed by the two invasive cell lines MDA-MB-231 and MDA-MB-468 by 57% and 70% respectively, compared with vehicle-treated controls (Figure 2B). Colonies were also smaller in size in the antibody treated samples. The MCF10A cells did not form colonies in soft agar (remained as single viable cells), as expected, and were not affected by the presence of the antibody. For the Matrigel assay, four different cell lines with distinct 3D morphologies (MCF10A—round; MCF-7—mass; MDA-MB-468—grape-like; and MDA-MB-231—stellate) were plated onto Matrigel and grown for 7 days. Medium ± H200 Ab was replaced every 3–4 days. MCF10A cells formed small colonies of cells and appeared unaffected by the presence of the antibody (Figure 2C—panel 1). Untreated MCF-7 cells formed large masses of cells but the sizes of the masses were significantly reduced by addition of the antibody (Figure 2C—panel 2). MDA-MB-468 form sparse grape-like colonies on Matrigel and antibody treatment caused significant cell death (Figure 2C—panel 3). MDA-MB-231 cells initially formed colonies but after 7 days there was outgrowth of stellate colonies from these in the untreated cells that was not seen in the colonies treated with H200 antibody (Figure 2C—panel 4).

### H200 treatment causes down-regulation of the *EphB4* gene and loss of the EphB4 protein

Breast cancer cell lines MDA-MB-231, MCF-7 and T47D were grown to confluent monolayers and treated with dilutions of H200 antibody (ranging from 1/100 down to 1/10,000) for 65 h before total RNA was extracted for gene expression analysis. EphB4 gene expression was consistently less, to nearly absent, in some cells treated with antibody when compared to control untreated cells (Figure 2D). Western blot analysis of EphB4 protein levels in MCF-7 cells treated for 24, 48 or 72 h with 0.4 μg/mL H200 antibody showed that there was also a correlated reduction in EphB4 protein in these cells (Figure 2E). In triplicate experiments, the EphB4 protein level was significantly less than both control untreated cells and cells treated with a mouse IgG control for 72 h (Figure 2F).

### Mapping the polyclonal antibody using peptide exclusion assays

**Figure 3:**
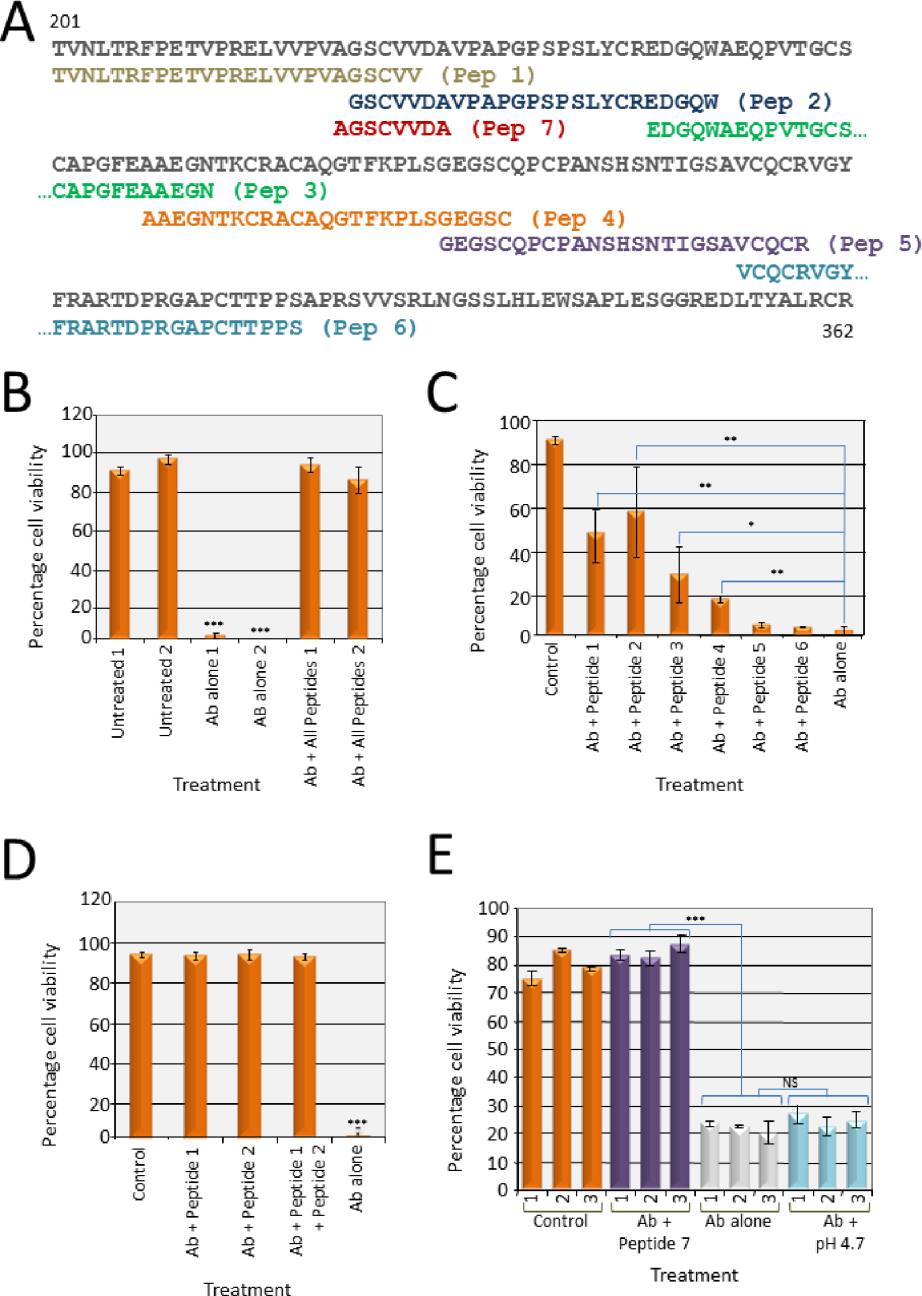
Peptide exclusion assays to map the critical epitope recognized by EphB4 H200 antibody **(A)** Six 25 amino acid peptides were designed to the first 125 amino acids of the 200 amino acid region (201–400) recognised by the H200 antibody and encompassing most of the cysteine rich region (184–320). Peptide 7 spans the overlap between peptides 1 and 2. **(B)** Cell viability was determined in the presence of H200 pAb pre-incubated with a mixture of peptides 1–6 before addition to the cells (Ab+ all peptides, 0.1 mg/ml each) and cell viability was determined using a trypan blue assay after 65 h. Viability of cells treated with the peptide blocked H200 pAb was consistent with the level of the control PBS-treated cells (untreated). There were no viable cells after treatment with the antibody alone (Ab alone) for 65 h (***p < 0.001). This experiment was performed in duplicate. **(C)** H200 pAb was pre-incubated with 0.1 mg/ml of each of the peptides 1–6 individually, as indicated, before addition to the cells. At 65 h, peptides 1 and 2 restored viability to 50% of the control PBS-treated cells (untreated). There were no viable cells after treatment with the antibody alone (Ab alone) (*p < 0.01, **p < 0.05, ***p < 0.001). **(D)** H200 pAb was pre-incubated with 0.1 mg/ml of each of the peptides 1 and 2, or 0.2 mg/ml Peptide 1, or 0.2 mg/ml Peptide 2 as indicated before addition to the cells. At 65 h, viability was restored to the level of the control PBS-treated cells (untreated). There were no viable cells after treatment with the antibody alone (Ab alone) (***p < 0.001). **(E)** H200 pAb was pre-incubated with 0.2 mg/ml Peptide 7 (spans the overlapping sequence common to Peptides 1 and 2). Viability was restored to the level of the control PBS-treated cells (untreated). There were no viable cells after treatment with the antibody alone (Ab alone). Because the peptide 7 solution was acidic and this may have negatively affected the antibody binding, antibody was also pre-treated with PBS adjusted to pH 4.7. Viable cells after antibody pH treatment was consistent with viability the antibody untreated (Ab alone) (***p < 0.001, NS = not significant).

The EphB4 polyclonal antibody was raised against a recombinant protein corresponding to amino acids 201–400 mapping within an extracellular domain region of human EphB4 (Figure 1A). The sequence includes the cysteine-rich domain and the first fibronectin type III repeat and accordingly it was possible that several different antigenic regions were recognized. We chose to explore the cysteine-rich region in the first instance and designed six, 25 amino acid peptides corresponding to this region for antibody competition studies (Figure 3A, peptides 1–6). Each peptide (0.1 mg/ml) was tested individually over 65 h in culture for potential toxic effects on cancer cell growth. None of the peptides caused morphological, cell growth or apoptotic changes (data not shown), indicating that the peptides themselves were not toxic to the cells.

To test whether these peptides could block the cell death function of the EphB4 H200 Ab, a cocktail of all peptides (0.1 mg/ml each) was pre-incubated with EphB4 antibody (0.4 μg/ml) for 1 h at room temperature before addition to a confluent monolayer of SW480 cells. No inhibitory Ab response was seen, even after 65 h in culture, indicating that one or more of the peptides was binding to the functional Ab in the polyclonal mix and preventing attachment to the native EphB4 protein expressed on the tumour cell surface (Figure 3B). Peptides (0.1 mg/mL) were then tested separately and the antibody blocking response was seen (approximately 50% of that seen using all of the peptides together) after the addition of either peptide 1 or 2 (Figure 3C). The other four peptides were less successful at preventing the cell death effect of the H200 Ab. The sequences of peptides 1 and 2 overlap by five amino acids—GSCVV (Figure 3A). Testing peptides 1 and 2 individually at 0.1 mg/mL would have effectively halved the concentration of the GSCVV sequence used in the first assay (0.2 mg/mL combined). Accordingly, a two-fold increase of either peptide 1 or peptide 2 (to 0.2 mg/mL) was sufficient to double the concentration of GSCVV in the test and thereby fully block the H200 Ab (Figure 3D) identifying GSCCV as the core of the epitope through which H200 functions to induce cell death.

To confirm that this was the key sequence, a further peptide, named peptide 7 comprising 8 amino acids with the sequence AGSCVVDA was produced (Figure 3A). Peptide 7 was confirmed to be non-toxic to cells (data not shown). This peptide used at 0.2 mg/ml effectively blocked the Ab response (Figure 3E). It was noticed that peptide 7 lowered the pH of the solution to pH 4.7 and because it was possible that this low pH might cause unfolding and therefore inactivation of the H200 Ab, in a control assay the pH of an H200 aliquot was lowered to pH 4.7 for 1 h before it was used to treat the cells. There was no change seen in the effectiveness of the Ab to decrease cell viability (Figure 3E).

### Peptide 7 selected EphB4-specific antibody behaves as an ephrin-B2 mimetic

**Figure 4:**
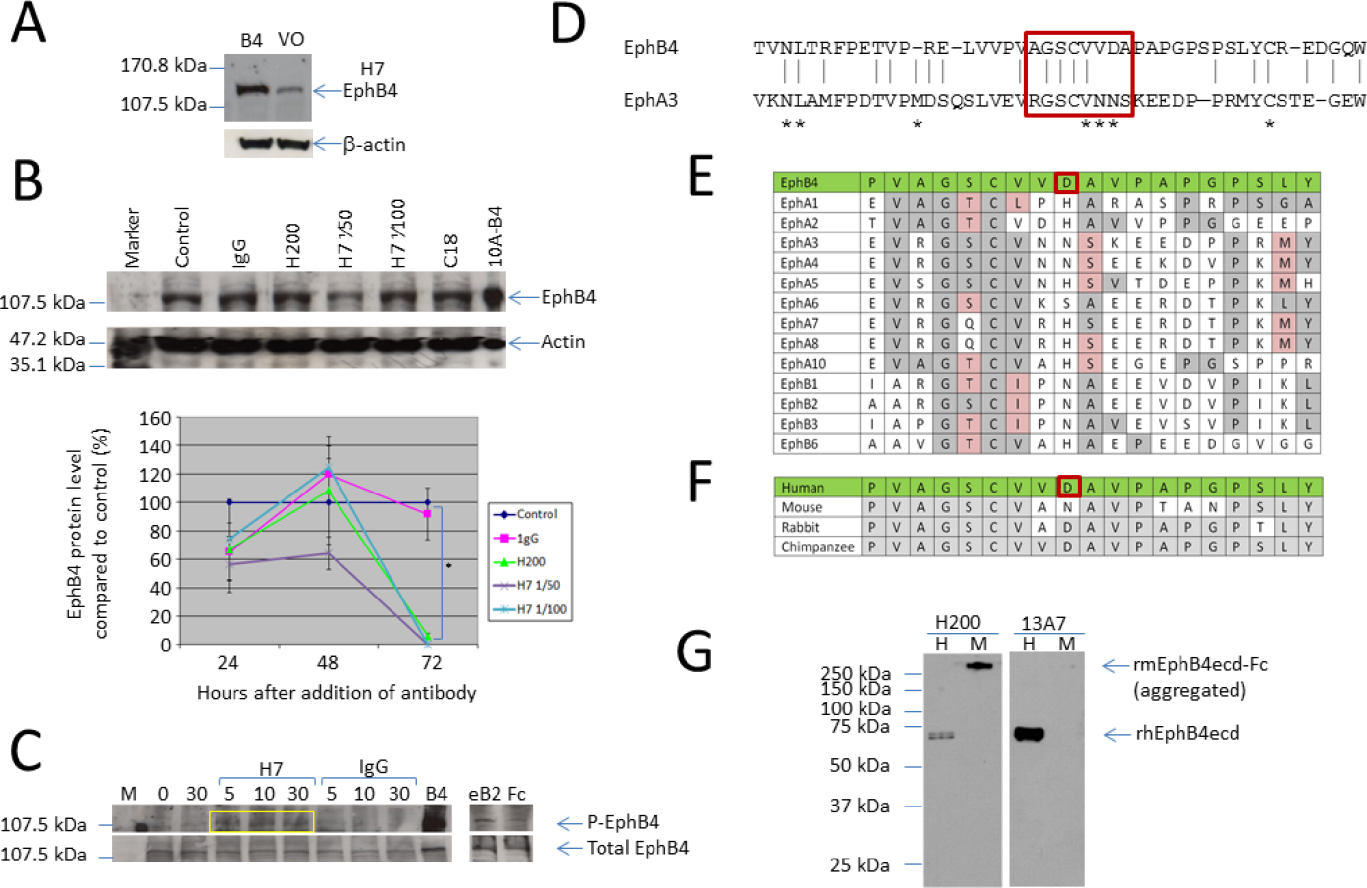
Peptide specific antibodies identify EphB4, cause EphB4 protein phosphorylation and degradation **(A)** Antibodies specifically recognizing Peptide 7 were isolated from the H200 polyclonal preparation. Western blot analysis shows that these antibodies (H7) specifically identify EphB4 with less non-specific cross-reactivity than the H200 antibody. β-actin was used as a loading control. **(B)** Western blot and graph showing that in a similar manner to H200, the H7 antibody, tested at two different dilutions, reduces total EphB4 protein level with a significant reduction seen at 72 h (*p* = 0.0001). **(C)** Immunoprecipitation of cell lysates from MCF-7 breast cancer cells showing treatment with the H7 antibody causes phosphorylation of the EphB4 protein, seen at 5 min after addition and maintained at 30 min (yellow box). Cells were stimulated with soluble clustered ephrin-B2-Fc ligand (eB2) as a positive control for phosphorylation with clustered Fc fragment only (Fc) as the negative control for this. **(D)** Alignment of the EphB4 sequence of peptides 1 and 2, which includes peptide 7 sequence AGSCVVDA (red box) with the corresponding sequence from EphA3. Asterisks identify the amino acids identified as contributing to the third ligand binding domain. **(E)** Comparison of the peptide sequence used to raise EphB4 monoclonal antibodies (green) with other members of the human Eph family. Amino acids identical to the EphB4 sequence are shaded in grey, homologous amino acids are shaded pink. The antigenic aspartic acid reside is within a red box. **(F)** Comparison of the peptide sequence used to raise human EphB4 monoclonal antibodies (green) with EphB4 sequences from other species. Amino acids identical to the human EphB4 sequence are shaded in grey. **(G)** Western blot analysis using the H200 and 13A7 antibodies with recombinant human EphB4 extracellular domain (rhEphB4ecd) (H) and recombinant mouse EphB4 extracellular domain Fc fusion protein (rmEphB4ecd-Fc) (M). The arrows indicate the position of the proteins compared to the molecular weight marker for which sizes are indicated on the left.

Peptide 7 was immobilized onto a MicroLink (gel matrix used to isolate interacting antibodies (designated H7) from the H200 polyclonal antibody preparation. To confirm that isolated antibodies could specifically identify EphB4 we again compared the MCF10A and MCF10A-B4 cells using Western blot analysis (Figure 4A). When compared with the H200 pAb, the H7 antibody detected only a single strongly immunoreactive band at the predicted 120 kDa, seen in the MCF10A-B4 sample and less strongly in MCF10A, consistent with detection of the over-expressed EphB4 in MCF10A-B4 and the endogenous EphB4 in MCF10A. Extended stimulation (for 72 h) led to a loss of EphB4 protein from the MCF-7 cells in a similar manner to that of the parental H200 antibody (Figure 4B and Figure 2F). Addition of the purified H7 antibody to MCF-7 cells caused an increase in EphB4 phosphorylation as soon as 5 min after addition which was still maintained at 30 min suggesting that the antibody activates EphB4 signaling in treated cells (Figure 4C). This would suggest that the antibody is behaving as an ephrin-B2 mimetic as ligand stimulation of EphB4 normally leads to phosphorylation of the kinase domain, then internalization and degradation of the receptor. In keeping with this hypothesis, the AGSCVVDA sequence contains three amino acids that align with those of a defined third ligand interface region on the EphA3 receptor (Figure 4D). Comparison with sequences of other Eph receptors shows variability at key residues suggesting high epitope specificity of the H7 antibody (Figure 4E). For example, analysis of the sequence using the EMBOSS antigenic prediction program (http://emboss.sourceforge.net/apps/release/6.0/emboss/apps/antigenic.html) identifies the aspartic acid residue in the EphB4 sequence (red box in Figure 4E) as the key amino acid within the most antigenic sequence in the 200 amino acid region targeted by the H200 antibody. Comparison with sequences from other species shows few differences in the amino acid sequence of the corresponding regions but the most differences to mouse EphB4 (Figure 4F) and Western blot analysis shows that although the H200 antibody can detect both human and mouse EphB4 proteins, the 13A7 antibody that targets the peptide 7 sequence specifically, only identified human EphB4 protein (rhEphB4ecd) and not murine EphB4 protein (rmEphB4ecd-Fc) (Figure 4G).

### Production and characterization of a panel of EphB4 monoclonal antibodies targeting defined epitope

**Figure 5:**
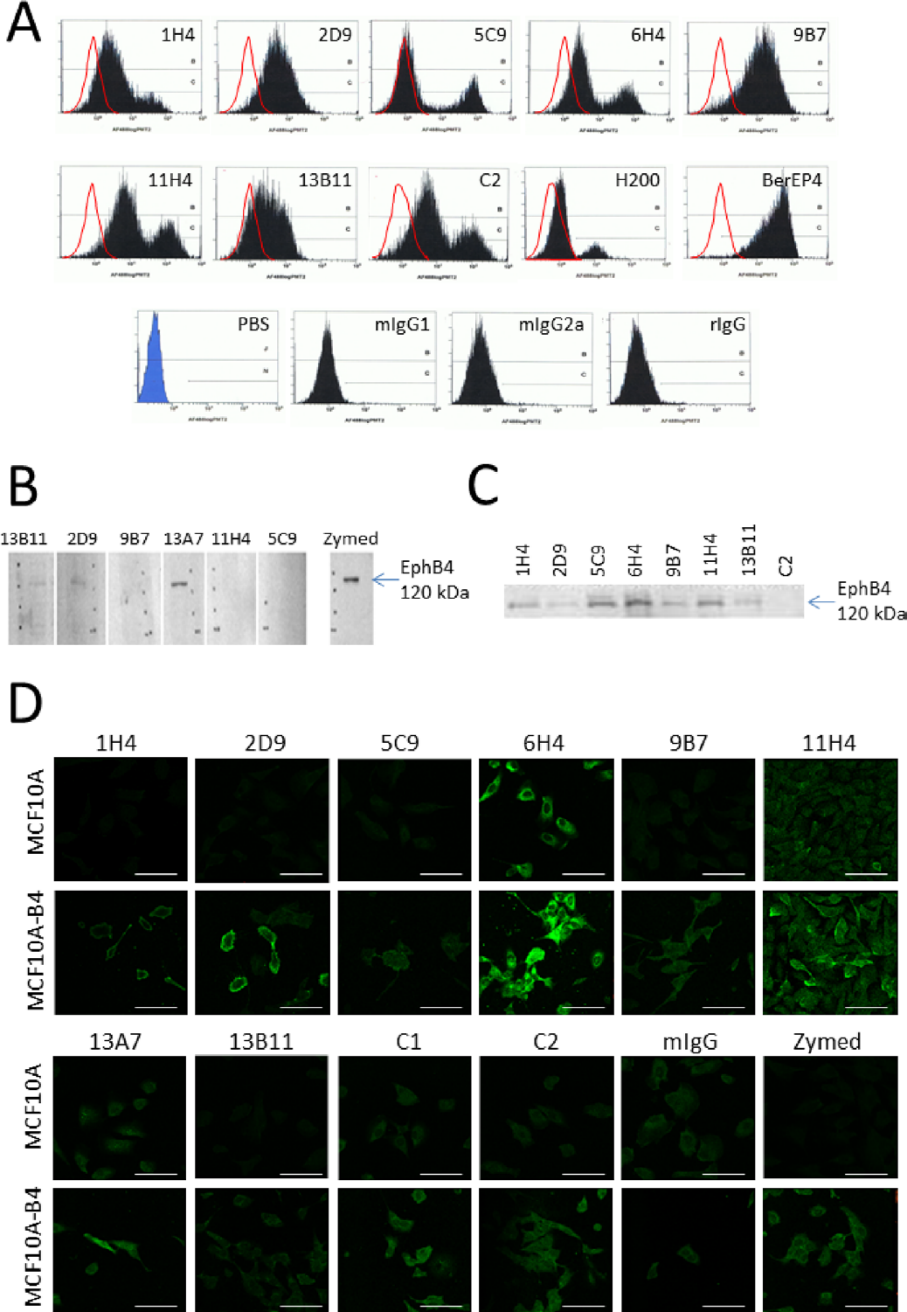
Validation of the EphB4-specificity of the epitope targeting monoclonal antibodies and their use for detecting EphB4 using different techniques **(A)** Monoclonal antibodies were screened using flow cytometry to detect EphB4 expression on MCF-7 cells. The shift in peak fluorescence to the right (black filled) is compared with the matched isotype control (mIgG1, mIgG2a or rIgG) (red line) and is due to antibody binding. The H200 was used for comparison and the BerEP4 antibody that detects the epithelial glycoprotein EpCam was used as the positive control. **(B)** Western blot analysis showing that denatured and reduced EphB4 (120 kDa) can be identified using antibodies 13A7, 13B11 and 2D9. The Life Technologies “Zymed” monoclonal antibody was used as the positive control. **(C)** EphB4 was immunoprecipitated from total protein lysates using several of the monoclonal antibodies. EphB4 was identified in the immunoprecipitated sample *via* Western blot analysis using the Zymed antibody. **(D)** Immunofluorescence comparing EphB4 detection (green) in the MCF10A and MCF10A-B4 cells using the monoclonal antibodies. Bar = 80 μm.

A panel of murine monoclonal antibodies was raised to the sequence AGSCVVDAVPAPGPSLY (Figure 4E). These antibodies were validated to the EphB4 protein using several methods. Firstly, each antibody was used to detect cell surface expression of EphB4 in MCF-7 cells by flow cytometry (Figure 5A). All antibodies caused an increase in fluorescence of the MCF-7 cells when compared with mIgG labelled cells (black peak shifted to right of clear peak) which shows that the antibodies are binding to the MCF-7 cells *via* the EphB4 expressed by these cells. Differences in the shape of the peaks, with some antibodies showing a single peak while others showed two peaks, may reflect heterogeneity of EphB4 expression levels and epitope presentation in the MCF-7 cell population, but also the affinities of the various antibodies. The BerEP4 antibody that recognises the epithelial EpCam glycoprotein was used as a positive control and mIgG1a, mIgG2a and rIgG antibodies were used as negative controls. Antibodies were then tested using Western blot analysis of MCF10A-B4 lysates. Three antibodies, 13B11, 2D9 and 13A7, were able to identify denatured and reduced EphB4 protein with 13A7 performing best in this application (Figure 5B). In a third validation experiment, antibodies were used to immunoprecipitate EphB4 from total MCF10A-B4 protein lysates and this was then detected by Western blot analysis using the Zymed antibody. Several antibodies successfully pulled down EphB4 confirming that maintenance of epitope folding is important for binding of some antibodies (Figure 5C). Finally, antibody recognition of EphB4 expressed on MCF10A and MCF10A-B4 cells was compared using immunofluorescence. A stronger green fluorescence signal was seen in MCF10A-B4 cells compared with MCF10A cells using several of the antibodies with 2D9, 6H4 and 11H4 performing particularly well (Figure 5D).

### Antibody C2 reduces growth of MDA-MB-231 cells

**Figure 6:**
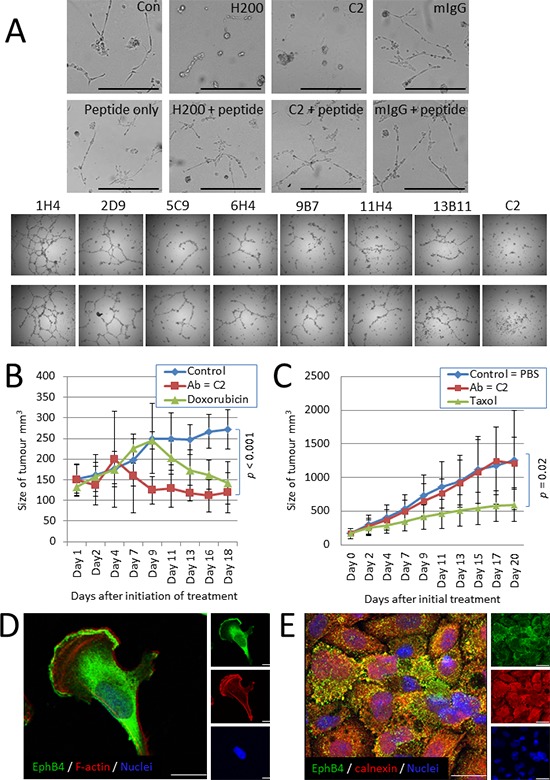
Antibody C2 is effective against MDA-MB-231 cells with surface EphB4 expression **(A)** Each antibody was tested for their ability to prevent tubular network formation of MDA-MB-231 cells grown on Matrigel. C2 was the most effective in duplicate wells. **(B)** Tumour growth data for MDA-MB231 xenograft tumours treated daily for 18 d with 50 mg/kg anti-EphB4 antibody C2 (red squares), positive control chemotherapeutic Doxorubicin™ (green triangles) or untreated (PBS) control group (blue diamonds). The C2 monoclonal antibody significantly reduces the size of tumours by 18 days (*p* < 0.001) and even performs better than Doxorubicin™ over this time course. **(C)** Tumour growth data for PC3 xenograft tumours treated daily for 18 d with 50 mg/kg anti-EphB4 antibody C2 (red squares), positive control chemotherapeutic Taxol (green triangles) or untreated (PBS) control group (blue diamonds). The C2 antibody does not affect PC3 tumour growth. **(D)** Immunofluorescence localization of EphB4 (green) in MDA-MB-231 cells using the C2 antibody. Phalloidin-TRITC (red) stains F-actin, DAPI (blue) stains the nucleus. Bar = 10 μm. **(E)** Immunofluorescence localization of EphB4, using the C2 antibody (green), and the endoplasmic reticulum marker calnexin (red) in PC3 cells. DAPI (blue) stains the nucleus. Bar = 10 μm.

Cell based assays testing antibodies that recognise a native epitope of the target protein where the effect of the antibody on the growth of the cells can be determined are an important prelude to *in vivo* testing. Because EphB4 has been shown to be important to cancer cell migration and invasion, we screened our antibodies for their effect on the formation of cancer cell networks on Matrigel, a cell-based assay that recapitulates *in vivo* cell growth and particularly the ability of cancer cells to migrate and invade the extracellular matrix. In this assay, the C2 antibody best prevented network formation of MDA-MB-231 breast cancer cells in both duplicate wells with 1H4 showing a web-like network similar to that of the no antibody or mIgG controls (Figure 6A and data not shown). For this reason C2 was tested for its effect on the growth of established MDA-MB-231 murine xenografts. Based on acute tolerated dose (ATD) and maximum tolerated dose (MTD) studies performed by VivoPharm (Adelaide, Australia), the maximum dose of 50 mg/kg C2 antibody was chosen. After 18 days (with C2 treatment given every three days), MDA-MB-231 tumours showed a significant decrease in size when compared with control untreated tumours and this was comparable to the response of the tumours to the chemotherapeutic agent, Doxorubicin (Figure 6B). We also tested the PC3 prostate cancer cell line, which has been reported to over-express EphB4 endogenously and for which knockdown experiments have shown EphB4 expression is required for cancer cell viability, migration and invasion [6]. The PC3 tumours did not respond to the C2 antibody (Figure 6C). Immunofluorescence using the antibody C2 to visualize EphB4 in MDA-MB-231 and PC3 cells shows that the C2-responsive MDA-MB-231 cells express EphB4 on the surface and in the cytoplasm (Figure 6D) but the PC3 cells show little surface expression with most of the C2-recognising EphB4 co-localising with the endoplasmic reticulum marker calnexin (Figure 6E). This highlights the need for complementary development of a screening technique that can accurately identify patients with high level surface expression of EphB4 and therefore those most likely to benefit from a monoclonal antibody therapy targeting EphB4.

## DISCUSSION

Monoclonal antibody therapies are proving useful in the treatment of diseases including cancer but the success of these depends on the identification of suitable target proteins that play important roles in cancer progression by contributing to the hallmarks of cancer and are homogenously over-expressed and accessible on the surface of tumour cells [43]. EphB4 meets all of these characteristics in many cancer cell lines used as models of different epithelial cancers making it an ideal target for the development of new monoclonal antibody-based anti-cancer therapies. To date, EphB4 has been shown to be over-expressed in 1046 of 1318 (82%) individual tumour samples from a wide selection of epithelial cancers. Knockdown experiments using cell lines representing several of these epithelial cancers have shown that EphB4 increases cancer cell viability, and contributes to migration and invasion [6, 11, 26–27]. Over-expression studies have confirmed this, showing that increasing EphB4 levels can confer a transformed phenotype (breast MCF10A) and an increased metastatic phenotype (prostate cancer 22Rv1) [28]. Importantly, both phenotypes can be restrained by stimulation with ephrin-B2 proving that over-production of EphB4 contributes in a ligand-independent mechanism and that ligand-dependent stimulation is tumour suppressive.

It is this dichotomy of EphB4 signaling that makes this receptor tyrosine kinase a unique target for the development of anti-cancer therapies. Successful approaches will use therapies that 1) mimic the ephrin-B2 ligand and stimulate tumour suppressive signaling pathways, 2) cause internalisation and degradation of EphB4 preventing further ligand-independent signaling that is tumour promoting and 3) can block further *de novo* EphB4 production. This is in clear contrast to approaches used to target other receptor tyrosine kinases such as EGFR and HER2, where prevention of receptor interaction with ligand (soluble growth factors) or inhibition of tyrosine kinase activity, using small molecules that sit in the ATP binding pocket, are proving successful [44–45]. It is possible that “leaky” tyrosine kinase inhibitors may also interact with EphB4, blocking the tumour suppressive signaling pathways and this may contribute to resistance seen in tumours that co-express EphB4 with the intended target receptor [46–47].

Phosphorylation and degradation of EphB4 in response to H200 addition suggest that the antibody can mimic the actions of the ephrin-B2 ligand. The mapped epitope sequence AGSCVVDA corresponds to amino acids 220 to 227 in the EphB4 extracellular domain and is at the N-terminal of the cysteine-rich region. A study by Smith et al (2005) shows that the N-terminal sequence of the cysteine-rich region contains a novel Eph/ephrin interaction interface [48]. Amino acid residues targeted by mutations within the region 208 to 232 of EphA3 define this third interface. A comparison of the amino acid sequences from EphA3 and EphB4 show that the sequence of the third Eph/ephrin interface falls entirely within the sequence defined by the overlapping EphB4 peptides 1 and 2. Furthermore, two of the amino acids targeted by mutations to define the EphA3 interface, V231D and N232I, are within the 8 amino acid sequence that is defined as the epitope through which the EphB4 H200 antibody acts to cause tumour cell death. This provides further support for our hypothesis that the H200 functional antibody mimics the ligand.

Addition of H200 to cancer cells *in vitro* causes a decrease in *EphB4* gene expression identifying a negative feedback loop. Knockdown experiments using EphB4 targeting siRNA sequences have shown that EphB4 contributes to cancer cell viability, migration and invasion identifying the key roles for EphB4 in cancer cells. In a manner consistent with this, H200 stimulation decreases cancer cell numbers and this correlates with an increase in caspase-3 activity indicating activation of apoptosis. H200 stimulation also prevents colony formation in soft agar and the formation of tubular networks on Matrigel, assays providing an *in vitro* indication of a negative effect of H200 on migration and invasion ability. This shows that the H200 antibody targets the hallmark roles EphB4 plays in cancer, justifying use of monoclonal antibodies for the development of new therapies against EphB4 positive cancers.

It is interesting that the H200 antibody only caused *in vitro* cell death after the cells had grown to a confluent monolayer. A similar response was reported by Carles–Kinch et al (2002) who identified an EphA2 mAb that recognizes a binding site present only in EphA2-positive cancer cells and reverses metastatic behaviour by activating the high levels of EphA2 on a tumour cell surface [49]. A coincident growth inhibitory effect was also only noticed after the cells had reached confluence. In both of these cases it would appear that a further requirement for this response is close proximity between neighbouring cells. Treatment with the H200 antibody caused the confluent monolayer of cells to lift from the dish as a fragile sheet. This shows that although the cells lost attachment to the dish, they maintained weak cell to cell attachment. Noren et al (2009) report that EphB4 kinase activity inhibits integrin-dependent cell adhesion [2]. Although not determined here, it is possible that a consequence of treatment with H200 is stimulation of EphB4 signaling pathways that alter integrin expression and/or activity. Recently Lehtinen et al (2013) reported that EphB4 silencing correlated with reduced expression of the intermediate filament protein vimentin and suggest EphB4 is a novel regulator of vimentin expression [25]. Vimentin is often upregulated during cancer progression and plays a key role in the epithelial to mesenchymal transition and cell invasion [50]. H200-induced loss of EphB4 might also cause loss of vimentin and this might contribute to the resultant decrease in invasion.

Our results show that a monoclonal antibody, designed to target the sequence mapping to a possible third ligand interacting domain within the cysteine-rich region of EphB4, and functioning as a ligand mimetic, can reduce/inhibit the *in vivo* growth of cancer cells that express EphB4 on the cell surface. This is consistent with reports from the literature that describe anti-cancer effects of EphB4 antibodies targeting other epitopes [37]. For example, two antibodies, MAb131 and MAb47, targeting separate epitopes in the first and second fibronectin type III (FIII) repeats of EphB4 respectively, have been developed and tested with some success *in vivo* using several different models of epithelial cancers [37]. The mechanisms through which these antibodies function is unlikely to be as direct ligand mimetics as FIII repeats are thought to have roles in protein-protein interactions especially in the extracellular matrix [51]. MAb131 can induce EphB4 degradation and inhibits endothelial tube formation *in vitro* and also inhibits the growth of EphB4-positive tumour xenografts [37]. Contrastingly, MAb47 had no effect on the level of EphB4 and inhibits the growth of both EphB4-positive and EphB4-negative xenograft models which suggests this antibody does not specifically target EphB4 but is instead functioning through another FIII-containing tumour protein [37].

With roles in regulating/modifying the important cancer progression hallmarks of viability, migration and invasion and common over-expression in up to 82% of epithelial cancers, EphB4 is a key target for the development of anti-cancer therapies. We demonstrate here that antibodies that target a ligand binding epitope cause phosphorylation and degradation of EphB4 in a ligand-mimetic manner and may prove particularly effective.

## METHODS

### Cell culture and antibodies

Cell lines used in this study were purchased from the American Type Culture Collection (ATCC) (Rockville, MD) and were cultured according to the ATCC recommendations in media and additives purchased from Life Technologies (Victoria, Australia). Three commercially available human EphB4-specific polyclonal antibodies EphB4 (N-19), EphB4 (H-200) and EphB4 (C-16) were purchased from Santa Cruz Biotechnology (Dallas, TX). An additional rabbit polyclonal antibody (S) recognizing human EphB4 (C-terminal) was a gift from Dr Andrew Ziemiecki, University of Bern, Switzerland [52]. For immunoprecipitation and Western blot analysis the C-terminal EphB4-specific mouse monoclonal antibody (Zymed, Life Technologies) and the phosphotyrosine specific 4G10 antibody (EMD Millipore, Billerica, MA) were used. The anti-human actin murine monoclonal antibody was from Abcam (Cambridge, MA). Secondary antibodies included a peroxidase-conjugated goat anti-mouse secondary antibody (Pierce, Thermo Fisher Scientific, Waltham, MA), a donkey anti-rabbit secondary antibody (Pierce) and Alexa Fluor^®^ 488 or Alexa Fluor^®^ 568, goat anti-mouse IgG (H + L) or goat anti-rabbit IgG (H + L) antibodies (Life Technologies).

### RNA isolation and relative quantitation of EphB4 expression

RNA was isolated from cell lines using Trizol™ solution according to the manufacturer's recommendations (Life Technologies) and quantitated using a Nanodrop Spectrophotometer (Thermo Fisher Scientific). Total RNA was reverse transcribed using Superscript III reverse transcriptase (Life Technologies). Primers specific to *EphB4* [8] and the housekeeping gene porphobilinogen deaminase (*PBGD)* [7] were used to PCR amplify gene-specific fragments from cDNA made using total RNA extracted from the various cell lines. PCR reactions were performed in a PTC-200 thermocycler (Perkin Elmer, Waltham, MA) with the following conditions: 2 μl cDNA template, 1.5 mM MgCl_2_, 200 μM each dNTP, 50 ng of each forward and reverse primer, and 0.5 units of Hot Star Taq polymerase in 1× PCR buffer (Qiagen, Limberg, Netherlands). Cycling conditions included an initial denaturation at 95°C for 15 min, followed by 40 cycles of 95°C for 30 sec, 58–68°C for 30 sec and 74°C for 30 sec, with a final extension of 74°C for 7 min. Amplification products were visualised by ethidium bromide staining following separation by electrophoresis through 1% agarose gels.

### Protein isolation, SDS-PAGE, western blotting and immunoprecipitation

Protein was isolated by direct lysis of cells grown in culture using RadioImmuno-Precipitation Assay (RIPA) buffer (15 mM HEPES, pH 7.4, 2% Triton X-100, 145 mM NaCl, 0.1 mM MgCl_2_, 10 mM EGTA, 1 mM sodium orthovanadate, 1 mM PMSF, aprotinin) supplemented with Complete protease inhibitors (Complete Mini-EDTA Free, Roche, Mannheim, Germany). Protein concentrations were determined using a BCA assay. Immunoprecipitation was performed using 4 μg antibody and Protein G Sepharose using the Immunoprecipitation Starter Kit and the manufacturer's instructions (GE Healthcare, Rydalmere, NSW). Proteins were separated using 10% SDS–PAGE gels then transferred onto BioTrace™ NT nitrocellulose membrane (Pall, Pensacola, FL). Membranes were blocked with Western blocking reagent (Roche) before incubation with primary antibodies to EphB4 including H200 (1:1000) and Zymed (1:1000) and to actin (1:10,000). Immunoreactivity was detected using the Amersham™ ECL Plus Chemiluminescence kit (GE Healthcare) and exposed to SuperRX X-ray film (Fuji Film Corporation, Japan).

### Cell viability assay using trypan blue exclusion

Cells were grown to full confluence before the addition of antibodies and incubation at 37°C for specified times ranging from 5 min to several days. The percentage of viable to non-viable cells was determined using trypan blue exclusion and light microscopy. Adherent cells were collected from the culture vessels using trypsin and combined with medium containing detached cells before all cells were collected using centrifugation at 1000 rpm for 5 min. The cell pellet was resuspended in 0.2% Trypan blue (Sigma, St Louis, MO) in PBS and after 5 min, the number of viable (unstained) and non-viable (stained) cells were counted using a haemocytometer.

### Apoptosis and cytotoxicity assays

Caspase-3 activity was determined as an indicator of apoptosis. Detached cells were washed from plates with 500 μl PBS and collected by centrifugation. Adherent cells were lysed using a buffer containing 5 mM Tris HCl pH 7.4, 5 mM EDTA and 5% NP-40 and the lysate removed to the tube containing the corresponding cell pellet. Samples were frozen at –20°C until needed for processing. A 50 μl aliquot of the cell lysis was added to 1 ml of caspase substrate buffer containing 50 mM HEPES, 10% sucrose and 0.1% CHAPS with 10 mM DTT and 1 μl/ml of the caspase-3 fluorescent substrate Ac-DEVD-AFC (Calbiochem, EMD Millipore). Reactions were incubated at room temperature overnight prior to measurement of caspase activation using a spectrofluorimeter with excitation at 400 nm and emission at 505 nm. Apoptosis was also measured by Annexin-V and propidium iodide staining followed by flow cytometry using an Annexin-V FLUOS kit (Roche). Lactate dehydrogenase (LDH) release, as a measure of cytotoxicity, was determined using the Cytotox 96^®^ Non-Radioactive Cytotoxicity Kit (Promega, Madison, WI).

### Assay of anchorage independent growth using soft agar

Colony formation in soft agar was performed as described by Clark et al (1996) [32]. Cells were resuspended in a molten solution of 0.3% agar (Merck, EMD Millipore) with the appropriate medium, depending on the cell line being used for the assay, and set onto a 0.8% medium-agar base. Antibody (10 μg/ml) or PBS vehicle was included in the top agar solution. Colony formation after 12 days was visualised microscopically using a Nikon Eclipse E800 light microscope at 20× magnification and images recorded using a Diagnostic Instruments Camera Model 2.3.1 and Image-Pro Plus 4.1 for Windows.

### Assay of network formation on Matrigel

The growth of cells on Matrigel was analyzed as described in Giunciuglio et al (1995) [33]. Briefly, 260 μl of 10 mg/ml Matrigel (BD Biosciences, Bedford, MA) was polymerized in each well of a 24-well tissue culture plate for 30 min at 37°C. The matrigel layer was then dehydrated overnight and re-hydrated 30 min before use with 260 μl of cell culture medium. Excess medium was carefully removed before the addition of 5 × 10^4^ cells with either the H-200 antibody (10 μg/ml) or vehicle (PBS). Cells were incubated on Matrigel for 72 h under normal tissue culture conditions and network formation was assessed using a Nikon Eclipse E800 light microscope and recorded digitally using a SciTech camera (Melbourne, Australia). Images were processed using SPOT Advanced Software Version 3.5.

### Epitope identification using a peptide exclusion approach

Six, consecutive 25 amino acid peptides that overlapped by five amino acids and corresponded to amino acids 201–326 of the extracellular domain of EphB4 were manufactured commercially by Auspep (Sydney, Australia). These were reconstituted to a stock solution of 10 mg/ml for use at a range of concentrations from 0.1 to 0.2 mg/ml. Peptides were pre-incubated with 0.4 μg/ml EphB4 H-200 antibody in DMEM for 1 h at room temperature before addition to confluent monolayers of cells. Cells were grown under normal culture conditions for up to 72 h before collection for viability and apoptosis assays.

### Peptide affinity purification of antibody

A peptide corresponding to the mapped epitope (Peptide 7) was made commercially by Auspep. This was coupled to a gel matrix using the MicroLink™ Peptide Coupling Kit (Pierce) and the manufacturer's instructions then used to purify antibodies that bound to the epitope from the H200 polyclonal antibody mix.

### Immunofluorescence

Immunofluorescence was performed using the method reported in Rutkowski et al (2012) (28). Briefly, 1 × 10^5^ cells were seeded onto cover slips and allowed to attach for 24 h before being fixed using 4% paraformaldehyde and permeabilised using 0.3% Triton X-100. Non-specific binding was blocked using 10% goat serum before addition of antibodies overnight at 4°C. After washing, bound primary antibody was detected using a secondary antibody conjugated to a fluorescent tag. In some experiments phalloidin conjugated to tetramethyl rhodamine isothiocyanate (TRITC) (Sigma, St. Louis, MO) was used to stain F-actin. Cover slips were mounted on glass slides using Prolong Gold Antifade with 4,6-diamidino-2-phenylindole DAPI (Life Technologies) and cells were visualised using a Leica SP5 spectral scanning confocal microscope. To identify EphB4 specific staining, control samples included one to which an irrelevant mouse IgG was added, another to which the secondary antibody alone was added and a third to which no antibody was added.

### Production of monoclonal antibodies

Five 6–8 week old Balb/c mice were immunized three times with a 21 amino acid containing the target epitope PVAGSCVVDAVPAPGPSPSLYK conjugated at the C-terminus to keyhole limpet hemocyanin (KLH). The first immunisation was performed using Freund's complete adjuvant and the others with Freund's incomplete adjuvant. Antibody titre was determined using a sample of blood collected by an incision of the tail vein and using an ELISA based assay for immunoreactivity to the peptide as described below. Hybridomas were produced by PEG-mediated fusion of splenocytes of the mouse with the highest titre with SP2/0 myeloma cells and selected in hypoxanthine-aminopterin-thymidine (HAT) medium. Eight individual monoclonal populations of hybridomas (1H4, 2D9, 5B7, 6H4, 9B7, 11H4, 13A7 and 13B11) each producing an antibody specific to the peptide, were selected by ELISA (method described below). Two further hybridomas (C1 and C2) were produced commercially (Millipore) using the same immunogen. All hybridomas were maintained in BD Cell™ MAb Medium Quantum Yield (BD Biosciences) and antibodies were purified from the medium by MAbSA (Adelaide, Australia).

### Enzyme-linked immunosorbent assay

Peptides, recombinant proteins or control solutions were used to coat the bottom of wells of 96-well Nunc Maxisorp ELISA plates (eBioscience, San Diego, CA). Non-specific binding sites were blocked using 5% bovine serum albumin (BSA) before the addition of hybridoma supernatant or antibody solution, and incubation overnight at 4°C. Bound antibodies were detected using a peroxidase-coupled secondary antibody, added after washing for 1 h at room temperature. The substrate *o*-Phenylenediamine dihydrochloride (SIGMA*FAST*™ OPD) was then converted by peroxidase activity to a coloured product that is measured at 492 nm in the presence of urea using an xMark™ Microplate Absorbance spectrophotometer (BioRad).

### Flow cytometry

Cells were removed from the dish with Versene and washed twice with fresh medium before counting using a haemocytometer. Cells were resuspended at 1 × 10^5^ cells/mL in Binding buffer (0.5 M HEPES, pH 7.4; 2% fetal calf serum; 2 μg/mL IgG) and 8 μg of primary antibody added before incubation on ice for 1 h. Cells were washed twice in cold Binding buffer before the addition of 0.5 μg of goat anti-mouse-FITC secondary antibody and incubation on ice for 1 h. Cells were again washed twice in ice-cold Binding buffer and the pellet resuspended in 600 μL of FACS Fix solution (1% paraformaldehyde and 1% glucose in PBS). Analysis of the cells was performed using a Beckman Coulter FC500 MPL Flow cytometer.

### *In vivo* tumour growth studies

Testing of the C2 antibody using human xenograft tumour models of breast cancer (using MDA-MB-231 cells) and prostate cancer (using PC3 cells) were performed by *vivo*Pharm (Adelaide, Australia). Initially, the acute tolerated dose (ATD) of the C2 antibody was determined using four groups of four mice, treated with 50 mg/kg, 20 mg/kg, 7 mg/kg and 0 mg/kg antibody (PBS vehicle only). Mice were observed for 7 days for signs of weight loss and changes to behavior. The maximum dose of C2 for which no effects were noted was 50 mg/kg and this dose was then used to determine the maximum tolerated dose (MTD). In this test, four mice in four groups were given either the maximum dose of 50 mg/kg C2 antibody (as per the ATD test), 80% of that dose (40 mg/kg), 60% of that dose (30 mg/kg) or remained untreated (PBS vehicle only). The mice were treated with this dose of C2 every day for 7 days and were observed for any negative effect. The maximum dose for which no effects are noted (50 mg/kg) was then used for testing the efficacy of the C2 antibody against xenograft tumour growth in mice. MDA-MB-231 or PC3 cells were implanted subcutaneously into the right flank of athymic (nu/nu) mice and tumours allowed to establish until they reached a volume of 80–120 mm^3^ before treatments began. Treatments included C2 antibody at 50 mg/kg, doxorubicin at 1.91 mg/kg as a positive control chemotherapeutic against MDA-MB-231 tumours, and Taxol at 10 mg/kg as a positive control chemotherapeutic against PC3 tumours. Each treatment was given three times weekly by intraperitoneal injection. Subcutaneous tumours were measured twice weekly using Vernier calipers and the tumour volume calculated using the formula V = L × W^2^/2, where V = volume, L = length and W = width. Mice were euthanized at the end of the experiment or when tumours grew > 1000 mm^3^ by cervical dislocation. Experiments were performed with animal ethics approval from the University of Adelaide, Australia.

### Statistical analysis

All results are expressed as mean ± SD. Differences between experimental groups were analysed by unpaired Student's *t*-test. *p* values < 0.05 were considered statistically significant.
